# Frequency of the requirement of inappropriate uncuffed tracheal tube size for pediatric patients: a retrospective observational analysis

**DOI:** 10.1186/s12871-021-01258-0

**Published:** 2021-02-03

**Authors:** Hiroshi Hanamoto, Hikaru Nakagawa, Hitoshi Niwa

**Affiliations:** Department of Dental Anesthesiology, Osaka University Graduate School of Dentistry, 1-8 Yamada-Oka, Suita, Osaka, 565-0871 Japan

**Keywords:** Intubation, Leak test, Leak pressure, Tidal volume, Uncuffed endotracheal tube

## Abstract

**Background:**

The insertion of inappropriately sized uncuffed endotracheal tubes (ETTs) with a tight seal or presence of air leakage may be necessary in children. This study aimed to analyze the frequency of the requirement of inappropriately sized uncuffed ETT insertion, air leakage after the ETT was replaced with one of a larger size, and factors associated with air leakage after ETT replacement.

**Methods:**

Patients under 2 years of age who underwent oral surgery under general anesthesia with uncuffed ETTs between December 2013 and May 2015 were enrolled. The ETT size was selected at the discretion of the attending anesthesiologists. A leak test was performed after intubation. The ETT was replaced when considered necessary. Data regarding the leak pressure (P_Leak_) and inspiratory and expiratory tidal volumes were extracted from anesthesia records. We considered a P_Leak_ of 10 < P_Leak_ ≤ 30 cmH_2_O to be appropriate. The frequencies of the requirement of inappropriately sized ETTs, absence of leakage after ETT replacement, ETT size difference, and leak rate were calculated. A logistic regression was performed, with P_Leak_, leak rate, and size difference included as explanatory variables and presence of leakage after replacement as the outcome variable.

**Results:**

Out of the 156 patients enrolled, 109 underwent ETT replacement, with the requirement of inappropriately sized ETTs being observed in 25 patients (23%). ETT replacement was performed in patients with P_Leak_ ≤ 10 cmH_2_O; leakage was absent after replacement (P_Leak_ < 30 cmH_2_O) in 52% of patients (25/48). In the multivariate logistic model, the leak rate before ETT replacement was significantly associated with the presence of leakage after replacement (*p* = 0.021).

**Conclusions:**

Inappropriately sized ETTs were inserted in approximately 23% of the patients. The leak rate may be useful to guide ETT replacement.

## Background

Although uncuffed endotracheal tubes (ETTs) have been routinely used to treat children under the age of 8 years, recent clinical data and practice guidelines have challenged this use [1]. Cuffed ETTs are now being increasingly used to treat pediatric patients [2]. Microcuff ETTs are theoretically beneficial due to their high-volume, low-pressure, and distal cuff design [3, 4]; however, these benefits may be limited to neonates [1]. A systematic review reported that the benefits of the use of cuffed as opposed to uncuffed ETTs during general anesthesia in children remain unclear [5]. Moreover, although the use of Microcuff ETTs in children may significantly reduce the consumption of sevoflurane and other anesthetic gases during inhalational anesthesia [3], Microcuff ETTs are not readily available in most low- and middle-income countries [5], since their cost is up to six times that of uncuffed ETTs [3, 6]. Therefore, Microcuff ETTs have yet to be routinely used worldwide in clinical practice [7].

When uncuffed ETTs are used, a certain amount of air leakage is recommended at airway pressures between 20 and 30 cmH_2_O to avoid excessive pressure on the tracheal mucosa [8, 9]. When air leakage is not observed at the appropriate airway pressure, the selected ETT is considered to be too large. In contrast, when air leakage is observed at a low airway pressure (e.g., ≤ 10 cmH_2_O), the selected ETT is considered to be too small. In these situations, the uncuffed ETT is replaced with another ETT of a smaller or larger size, respectively. However, even after this replacement, the size of the new ETT is not always appropriate. In such cases, it may not be possible to insert an appropriately sized uncuffed ETT because commercially available ETTs are usually produced at internal diameter gradations of 0.5 mm. Thus, an inappropriately sized uncuffed ETT with a tight seal (i.e., no leakage even at pressures over 30 cmH_2_O) or one that allows greater air leakage may be required, which subsequently results in the risk of ischemic damage to the subglottic mucosa [1] and aspiration, respectively [5]. So far, the frequency of the requirement of inappropriately sized uncuffed ETT insertion and the prediction of airway leakage after ETT replacement have not been clarified.

The need for replacing the ETT is usually determined based on the air leak pressure (P_Leak_) [10, 11]; however, low reliability of the leak test has been noted previously [12]. We hypothesized that parameters such as the fraction of ETT leakage [13, 14] were better predictors of the presence or absence of air leakage after replacing the ETT than the leak pressure. The purpose of this study was to assess: (1) the frequency of the requirement of inappropriately sized uncuffed ETT insertion, (2) the frequency of the absence of air leakage after replacing an ETT with one that is a size larger, and (3) the factors associated with the presence or absence of air leakage after ETT replacement.

## Methods

### Study design

This study was approved by the Ethics Committee of Osaka University Graduate School of Dentistry (approval number: R1-E7, approved on June 13, 2019). The requirement for written informed consent was waived because of the retrospective nature of the study. Patients aged less than 2 years who underwent oral surgery under general anesthesia and required tracheal intubation with preformed uncuffed ETTs between December 2013 and May 2015 at our hospital were included in this study, and their anesthesia records were retrospectively analyzed. The exclusion criteria were incomplete data and an American Society of Anesthesiologists physical status (ASA PS) of 3 or 4.

### Anesthesia induction and ETT size selection

After anesthesia induction, rocuronium (0.6–0.8 mg/kg) was administered, and the patient’s trachea was intubated with a Mallinckrodt Oral Right Angle ETT (COVIDIEN, Dublin, Ireland) that was cuffless and had a Murphy eye. Although we had obtained a regression equation for the outer diameter (OD) of the ETT based on our previous data [15, 16], the ETT size on the first attempt was selected at the discretion of the attending anesthesiologist. The fit of the ETT was then judged based on air leakage. A leak test was performed after each tracheal intubation as follows: the respiratory pressure was gradually increased up to 35 cmH_2_O, while one or two senior anesthesiologists listened for an audible leak sound near the patient’s mouth. Subsequently, volume-controlled mechanical ventilation (Fabius™ Tiro, Dräger, Lubeck, Germany) was initiated, with a respiratory rate of 18/min, tidal volume of 10 ml/kg, inspiratory-expiratory ratio of 1:2, and positive end-expiratory pressure (PEEP) of 0 cmH_2_O. After the tube size, P_Leak_, and expiratory tidal volume measured by the flow sensor were manually recorded, the appropriateness of the ETT size was assessed without the use of throat packs. At our institution, an uncuffed ETT was usually considered to be of appropriate size for oral surgery when no air leaks were observed at approximately 15 cmH_2_O, to prevent intraoral blood from entering the trachea. If an air leak was observed at an inflation pressure of less than 15 cmH_2_O, an ETT of one size larger was usually chosen, at the anesthesiologist’s discretion. However, if resistance and no air leak were observed at 35 cmH_2_O during ETT insertion, the intubated ETT was replaced with an ETT of a smaller size. In cases with no resistance observed during ETT insertion, the original ETT was usually retained. However, these criteria were not always adopted, and the ETT used was the one judged as most clinically appropriate by the attending anesthesiologist.

### Variables

Data regarding the participants’ age (days), height (cm), weight (kg), sex, ASA PS, surgery time, anesthesia time, ETT size and type, P_Leak_, and expiratory tidal volume (TV_Exp_) were extracted from their anesthesia records (MetaVision, Fukuda Denshi, Tokyo, Japan). The tidal volume set on the ventilator was considered as the inspiratory tidal volume (TV_Insp_).

### Data analysis

Although the appropriate range of P_Leak_ varies between institutions, we considered 10 < P_Leak_ ≤ 30 cmH_2_O as the appropriate range in our study to improve its generalizability. However, the ETT was replaced even if the P_Leak_ met this requirement in some cases. Thus, we had to additionally consider that ETT replacement was not always based on the criteria for analysis. The following were calculated:

1. The frequency of the requirement of inappropriately sized uncuffed ETT insertion. The data of the cases where the P_Leak_ was not within the appropriate range and the ETT was not replaced were excluded from this calculation.
2. The frequency of the absence of air leakage after replacing the ETT with one of a larger size due to a P_Leak_ ≤ 10.
3. The leak rate in all ETT replacements, based on the following formula: 100× (TV_Insp_−TV_Exp_)/TV_Insp_ (%) [13, 14].
4. The size difference.

We previously [15] focused on the size of the uncuffed ETTs in patients younger than 2 years of age because Cole’s formula [internal diameter (mm) = 0.25 × (age in years) + 4], which is commonly used, cannot be applied in cases that require ETTs smaller than 4.0 mm. Therefore, we previously reported a regression formula for the OD based on age in days [calculated OD (OD_Cal_) = 0.00223 × age (days) + 4.88, R^2^ = 0.511] among 1035 patients younger than 2 years of age [15]. In this study, the OD was calculated based on the patient’s age (OD_Cal_); however, this method is not routinely used during clinical practice. Subsequently, the difference between the OD of the selected ETT and the OD_Cal_ (OD_Dif_) were calculated (OD_Dif_ = OD − OD_Cal_). Thus, the size difference was defined as the difference in the OD of the selected ETT and the OD calculated based on the patient’s age.

### Statistical analysis

Statistical analyses, excluding those for the variance inflation factor (VIF), were performed using JMP, version 12.0 (SAS Institute, Cary, NC, USA). The VIF was analyzed using EZR (Saitama Medical Center, Jichi Medical University, Saitama, Japan), which is a graphical user interface for R software (The R Foundation for Statistical Computing, Vienna, Austria). More precisely, this program is a modified version of the R commander that was designed for statistical functions frequently used in biostatistics [17].

Continuous variables are shown as mean ± standard deviation and categorical variables as absolute numbers and percentages. The P_Leak_, leak rate, and size difference before ETT replacement were considered as explanatory variables, while the presence of leakage after replacing the ETT with one of a larger size was considered as an outcome variable. Each explanatory variable was compared between cases with the presence and absence of a leak using an unpaired t-test. Both univariate and multivariate logistic regression analyses were performed. Additionally, the correlation coefficients of P_Leak_ and leak rate, P_Leak_ and size difference, and leak rate and size difference were calculated. Moreover, the VIF of each variable was calculated. A correlation coefficient > 0.9 or a VIF > 10 was considered to indicate a high correlation. The results of the logistic regression models were expressed as *p-*values, odds ratios with 95% confidence intervals, cut-off values if appropriate, and areas under the curve. A *p-*value of less than 0.05 was considered statistically significant.

## Results

**Fig. 1:**
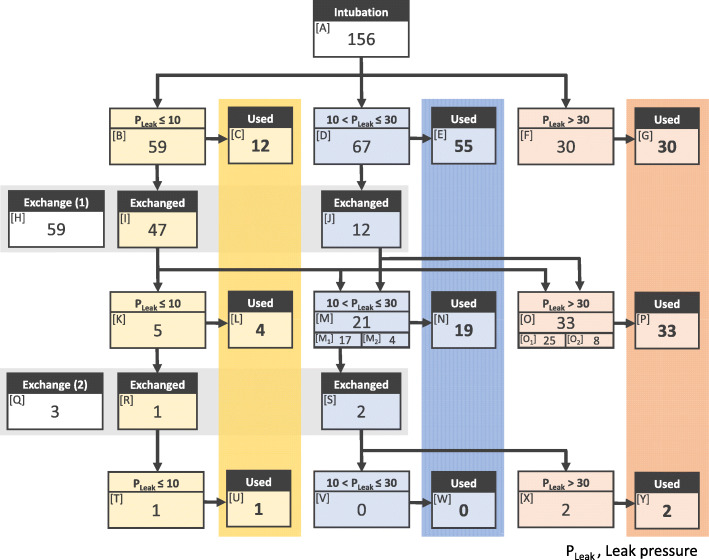
Patient flow chart showing their classification according to leak pressure. The endotracheal tubes inserted were divided into two categories: used or replaced. The number of patients in each group is shown. [M_1_] and [M_2_] are derived from [I] and [J], respectively. [M] is the sum of [M_1_] and [M_2_]. [O_1_] and [O_2_] are derived from [I] and [J], respectively. [O] is the sum of [O_1_] and [O_2_]

Of the 238 patients operated upon during the study period, 81 were excluded because of incomplete data, and one was excluded due to an ASA PS of 3. Hence, a total of 156 patients were included in this study. Their demographics are shown in Table 1. The classification of these patients, when an appropriate P_Leak_ was considered to be 10 cmH_2_O < P_Leak_ ≤ 30 cmH_2_O, is shown in Fig. 1.

**Table 1:** Patient demographics

Variable	Value
Age, Days	316 ± 185
Sex, *n* (%)	
Male	81 (52)
Female	75 (48)
Height, cm	70.2 ± 7.2
Weight, kg	8.0 ± 2.0
ASA PS	
1	142 (91)
2	14 (9)
Anesthesia time, min	137 ± 14
Surgery time, min	68 ± 23
Rocuronium, mg/kg	0.68 ± 0.09
Type of surgery, *n* (%)	
Cheiloplasty	64 (41)
Palatoplasty	90 (58)
Other	2 (1)

Values are mean ± standard deviation or number of patients (percentage)

*ASA PS* American Society of Anesthesiologists physical status

In 47 patients ([C], [G], [L], and [U] in Fig. 1), the selected tube was retained even though it did not meet the appropriate P_Leak_ criteria, and an additional tube replacement was not performed by the attending anesthesiologist, i.e., the attending anesthesiologist determined that the ETT size would likely be either too large or small if the ETT were replaced with a larger- or smaller-sized ETT, respectively, thereby prioritizing the reduction in the number of laryngoscopies. Therefore, in such cases, it was unclear whether an ETT that was the appropriate size for the patient was available. Thus, we assessed whether the ETT size satisfying the appropriate P_Leak_ criteria among the remaining 109 patients ([D], [M_1_], and [O_1_] in Fig. 1) were available. In some cases, the ETT was replaced at the discretion of the attending anesthesiologist, although it was theoretically unnecessary ([J] and [S] in Fig. 1). Consequently, an appropriately sized ETT was present in 84 patients ([D] and [M_1_] in Fig. 1, 77%) and absent in 25 patients ([O_1_] in Fig. 1, 23%).

ETT replacements were performed 62 times ([H] and [Q] in Fig. 1) in 59 cases. Of the 48 replacements ([I] and [R] in Fig. 1) in which an ETT replacement was performed due to a P_Leak_ ≤ 10 cmH_2_O, the air leak was absent < 30 cmH_2_O after replacement in 25 cases (52%).

**Fig. 2:**
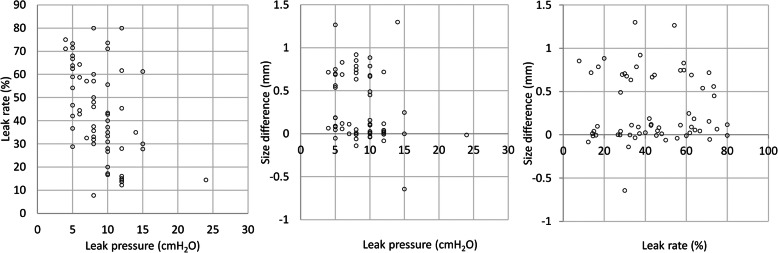
Correlations between the leak pressure, leak rate, and size difference

The comparison of the P_Leak_, leak rate, and size difference between the patients with- and without leakage is shown in Table 2. P_Leak_ and leak rate were significantly higher and lower, respectively, in the patients without leakage after ETT replacement (*p* = 0.045 and 0.002, respectively). The results of the univariate and multivariate logistic regression models for the presence or absence of leakage after ETT replacement are shown in Table 3. In the univariate logistic model, statistically significant differences were observed in P_Leak_ and leak rate (*p* = 0.034 and 0.002). Correlations between the explanatory variables are shown in Fig. 2. The correlation coefficients of the P_Leak_ and leak rate, P_Leak_ and size difference, and leak rate and size difference were − 0.477, 0.176, and 0.032, respectively. The VIF of P_Leak_, leak rate, and size difference were 1.23, 1.20, and 1.04, respectively. Thus, strong correlations of each explanatory variable were not observed. In the multivariate logistic model, the leak rate before ETT replacement was significantly associated with the presence of leakage after ETT replacement (*p* = 0.021).

**Table 2:** Leak pressure, leak rate, and size difference before endotracheal tube replacement

	With leak (*n* = 27)	Without leak (*n* = 35)	*p*-value
Leak pressure, cmH_2_O	7.8 ± 2.7	9.6 ± 3.9	0.045
Leak rate, %	52 ± 17	37 ± 19	0.002
Size difference, mm	−0.27 ± 0.46	−0.28 ± 0.40	0.900

Values are mean ± standard deviation

**Table 3:** Univariate and multivariate logistic regression models for the presence of leakage after endotracheal tube replacement

Variables	*p-*value	Odds ratio (95% CI)	AUC	Cut-off value
Univariate model				
Leak pressure	0.034	0.84 (0.69–0.99)	0.641	8
Leak rate	0.002	1.04 (1.02–1.08)	0.723	43.3
Size difference	0.898	0.92 (0.27–3.09)	0.502	0.011
Multivariate model				
Leak pressure	0.420	0.92 (0.75–1.12)	0.731	
Leak rate	0.021	1.04 (1.01–1.08)		
Size difference	0.844	0.87 (0.22–3.29)		

*AUC* area under the curve, *CI* confidence interval

## Discussion

The main findings of this study were that (1) appropriately sized ETTs were present in 77% of patients, (2) air leakage was absent at the pressure of 30 cmH_2_O after ETT replacement in 52% of patients, and (3) leak rate rather than P_Leak_ was associated with the presence of air leakage after ETT replacement.

Although there have been many studies [18–23] on the size of uncuffed ETTs that determined the appropriate size based on P_Leak_, most of these studies did not report the data on the inappropriately sized ETTs; this was done only by Park et al. [22] They investigated uncuffed ETT size among 605 patients between 3 and 6 years of age. After excluding patients based on their exclusion criteria, such as air P_Leaks_ of < 10 or > 30 cmH_2_O, they finally analyzed the data of 537 patients. In their study, appropriately sized ETTs were not available in 26 of 563 cases (4.6%). In the present study, appropriately sized ETTs were not available in only 25 of 109 cases (23%), which was approximately five times the frequency documented by Park et al. [22] This difference might be attributed to the age of the study population: 3–6 years in Park et al. vs. less than 2 years in this study. Generally, the difference in the inner diameter between each successive size is 0.5 mm. The proportion of change is larger in smaller-sized ETTs, even when the actual difference remains 0.5 mm. Therefore, our results suggest that uncuffed ETT size selection is difficult in younger patients.

In our study, of the 48 patients ([I] and [R]) in whom ETT replacement was performed due to a P_Leak_ ≤ 10 cmH_2_O, air leak was absent under 30 cmH_2_O after replacement in 25 patients (52%). However, this included patients with a P_Leak_ ≤ 10 cmH_2_O after ETT replacement. Therefore, air leak was absent in 60% (25/42) after the exclusion of these patients. This suggests that ETT replacement results in the absence of air leak in many cases. Therefore, we tried to predict the presence or absence of air leak after ETT replacement.

We compared the P_Leak_, leak rate, and size difference as indicators for the presence or absence of air leak after ETT replacement. All three parameters were included as explanatory variables in the multivariate logistic regression, since an extremely high correlation or VIF was not observed. In the multivariate logistic regression model, only the leak rate was statistically significant. This result suggests that the leak rate may be more useful as an indicator for ETT replacement than the P_Leak_.

Considerable variations in the assessment of P_Leak_ among different observers have been reported. Some observers felt that it was unreasonable to set an upper limit of P_Leak_ for ETT replacement. Further, a mean variance of 38% was observed in the mean P_Leak_ measured by two observers [12]. However, the appropriate size of the uncuffed ETT was still determined based on P_Leak_ in most of these studies [18–23].

We calculated the leak rate using the values of TV_Insp_ and TV_Exp_. In this study, TV_Exp_ was measured using a flow sensor; however, the ventilator setting value of TV_Insp_ was used, because the anesthesia machine had a flow sensor only on the expiratory side. Moreover, it is necessary to know the leak rate to reliably measure the respiratory function using a ventilator [13].

The upper and lower limits of the appropriate P_Leak_ vary between surgical procedures, hospitals, and anesthesiologists. Therefore, we used an equation formulated from the data from our hospital because we thought that it would fit our data in the best possible manner. However, the size difference was not a significant parameter for ETT replacement.

### Clinical implications

Since an ETT of the appropriate size was not initially inserted in 23% of patients, and air leakage was absent under 30 cmH_2_O after ETT replacement following a P_Leak_ ≤ 10 cmH_2_O in 52% of patients, we could infer that even when the ETT is replaced, a tube size that meets the appropriate P_Leak_ criteria will not always be available. Therefore, it is important to avoid unnecessary ETT replacement to reduce the number of laryngoscopies. Our study showed that the leak rate, rather than P_Leak_, was associated with the presence or absence of air leak after ETT replacement. Even if more accurate predictive parameters are available, they are not practical if they are difficult to measure. The leak rate can be calculated easily, and does not require additional equipment. The cut-off value in the univariate analysis was 43.3%. A leak rate of approximately 40% may be more useful than P_Leak_ to determine whether an ETT should be replaced with a larger one.

### Limitations

This study has several limitations, including the retrospective nature of the study and our utilization of a formula that is not recognized worldwide. In addition, there were errors in the measurement of the tidal volume using the ventilator included in the anesthesia machine [24]. According to the manufacturer, the measurement error of the flow sensor with which TV_Exp_ was measured within ±15%, and the instrumental error of the ventilator with which TV_Insp_ was measured within ±5%. Our results may have been influenced by these errors; however, to the best of our knowledge, a more accurate method of measurement does not presently exist.

Throat packs are sometimes used in clinical settings; however, there is controversy surrounding their risks and benefits [25]. The use of a throat pack varies depending on the institute and surgical procedure. In our hospital, although a throat pack is sometimes used during intraoral surgery, its use is not preferred during surgical procedures such as palatoplasty. Although the use of a throat pack may affect the ventilator parameters, our data were obtained without their use.

The effect of lung compliance and PEEP on the leak rate should also be considered. The use of PEEP is determined by multiple factors, including the type of surgery, institution, anesthetic machine, and attending anesthesiologist. PEEP is not commonly used at our institution and is not always applied in patients with uncuffed ETTs. Therefore, clinicians should be aware that the application of PEEP during the measurement of the leak rate might lead to a difference in the results. Moreover, the sample size was small in this study. Further, we could not continue this study prospectively, as we usually use Microcuff ETTs at our institution. A prospective study using uncuffed ETTs should be conducted at other institutions to confirm our findings.

## Conclusions

An appropriately sized ETT was not available in 23% of patients under the age of 2 who underwent uncuffed ETT insertion. Air leakage was absent under the pressure of 30 cmH_2_O after ETT replacement following a P_Leak_ ≤ 10 cmH_2_O in 52% of patients. Although a cut-off value could not be accurately determined using multivariate analysis, the leak rate rather than P_Leak_ was associated with the presence of air leakage after ETT replacement. Therefore, we suggest that anesthesiologists should measure the leak rate when using uncuffed ETTs.

## Data Availability

The datasets used in this study are available from the corresponding author on reasonable request.

## Abbreviations

ASA PS: American Society of Anesthesiologists physical status
ETT: Endotracheal tube
OD: Outer diameter
OD_Cal_: Calculated outer diameter
OD_Dif_: Difference in the outer diameter of the selected endotracheal tube and the calculated outer diameter
PEEP: Positive end-expiratory pressure
P_Leak_: Leak pressure
TV_Exp_: Expiratory tidal volume
TV_Insp_: Inspiratory tidal volume
VIF: Variance inflation factor
